# Remarkable Case of Constipation

**Published:** 1848-07

**Authors:** 


*Remarkable Case of Constipation.*—The following case is communicated in a letter to the editor of the *Boston Medical and Surgical Journal.* As the town where the patient is said to reside is in this neighborhood, we should be glad to have some farther particulars from some of our professional friends and subscribers residing on the spot.—Ed. Buff. Med. Jour.

Sir—While in Western New York last summer, I saw a remarkable case, in the person of a young lady, who has had but three fecal discharges in nine years; and thinking the statements in full might be interesting, if not useful, to the medical profession, I now communicate them.

The patient's name is Ellen R. Allis, daughter of Joel Allis. She lives about three miles from the centre of Batavia. When about twelve years of age, she attended a singing school, and being ensnared in a trap made by a rope by some boys, was thrown down, and her spine injured. She became quite sick, went to her bed, and there has remained up to the present time, perfectly bed-ridden. She is now twenty-one years old. She lays in her bed, her body being at an angle of forty-five degrees with her extremities; nor can she be removed from that position without producing intense pain, and much dyspnœa.

From her waist down there has been no increase in size since her first injury; but above the umbilical region, she has grown like other girls. She is wonderfully intellectual for one thus circumstanced, and has shown a remarkable skill in the formation of different curiosities.

As you would naturally conclude, she has but little physical strength. Her heart beats like an infant's, her respiration is peculiar, and so slight is the effort, that it is hardly distinguished by a careful observer.

Two days previous, and about three days after, the movements of her bowels occurred, she was perfectly insensible, and in a comatose state. About a teacupful of a thin viscid discharge come from her at each time.

There has been an attempt on the part of physicians in the region of Batavia to remove this difficulty, (whether it be a partial stricture of some portion of the intestines, or a want of action in them, is unknown,) but in vain.

Her diet is very light, consisting mostly of white sugar and tea, and occasionally a thin broth.

As you will understand, she has had but three stools in nine years. Oliver, in his Physiology, makes mention of some persons having no discharge in one year; but no instance like the above have I ever found recorded. Yours respectfully,

*Millbury, Mass., April* 13, 1848. CHARLES A. GREENE.

P. S.—If you wish any further information in reference to the above case, it may be obtained by directing a communication to me.

